# Intravenous lidocaine as a non-opioid adjunct analgesic for traumatic rib fractures

**DOI:** 10.1371/journal.pone.0239896

**Published:** 2020-09-28

**Authors:** Jeff Choi, Kirellos Zamary, Nicolas B. Barreto, Lakshika Tennakoon, Kristen M. Davis, Amber W. Trickey, David A. Spain

**Affiliations:** 1 Division of General Surgery, Department of Surgery, Stanford University, Stanford, CA, United States of America; 2 Department of Epidemiology and Population Health, Stanford University, Stanford, CA, United States of America; 3 Department of Surgery, St. Joseph Health Medical Group, Santa Rosa, CA, United States of America; 4 Stanford-Surgery Policy Improvement Research & Education Center, Department of Surgery, Stanford University, Stanford, CA, United States of America; Cleveland Clinic, UNITED STATES

## Abstract

**Introduction:**

Pain management is the pillar of caring for patients with traumatic rib fractures. Intravenous lidocaine (IVL) is a well-established non-opioid analgesic for post-operative pain, yet its efficacy has yet to be investigated in trauma patients. We hypothesized that IVL is associated with decreased inpatient opioid requirements among patients with rib fractures.

**Methods:**

We retrospectively evaluated adult patients presenting to our Level 1 trauma center with isolated chest wall injuries. After 1:1 propensity score matching patients who received vs did not receive IVL, we compared the two groups’ average daily opioid use, opioid use in the last 24 hours of admission, and pain scores during admissions hours 24–48. We performed multivariable linear regression for these outcomes (with sensitivity analysis for the opioid use outcomes), adjusting for age as a moderating factor and controlling for hospital length of stay and injury severity.

**Results:**

We identified 534 patients, among whom 226 received IVL. Those who received IVL were older and had more serious injury. Compared to propensity-score matched patients who did not receive IVL, patients who received IVL had similar average daily opioid use and pain scores, but 40% lower opioid use during the last 24 hours of admission (p = 0.002). Multivariable regression–with and without sensitivity analysis–did not show an effect of IVL on any outcomes.

**Conclusion:**

IVL was crudely associated with decreased opioid requirements in the last 24 hours of admission, the time period associated with opioid use at 90 days post-discharge. However, we did not observe beneficial effects of IVL on multivariable adjusted analyses; we are conducting a randomized control trial to further evaluate IVL’s opioid-sparing effects for patients with rib fractures.

## Introduction

Traumatic rib fractures confer significant morbidity and mortality. Poor respiratory effort limited by pain can result in pneumonia, respiratory failure, and ultimately, death [1–3]. This injury pattern is a growing problem: between 2006 and 2014, the incidence of rib fractures increased 19.4% despite a 12.9% decrease in emergency department visits for traumatic injury [4]. Rib fracture patients frequently require opioids for adequate pain management. However, known side effects of respiratory depression, the national spotlight on curbing opioid utilization, and risk of chronic opioid dependence after inpatient utilization have motivated physicians to investigate non-opioid adjunct analgesics [5–10]. Receipt of opioids during the last 24 hours of hospitalization may be an especially important measure, as this has been shown to be associated with opioid use at 90 days post-discharge [11]. Limiting opioid utilization is particularly critical for elderly patients (age ≥65 years) who are more vulnerable to side effects and are at higher risk of complications associated with rib fractures [12–14].

The efficacy of intravenous lidocaine (IVL) as an adjunct analgesic in rib fractures has not been investigated. Lidocaine is a widely used analgesic that is short-acting, easy to titrate, and has anti-inflammatory properties along with one of the best anesthetic safety profiles [15–17]. Even one dose of IVL has long-lasting analgesic effects, purportedly due to a continuous active biological response to lidocaine. Systemic IVL has been widely studied in surgical literature. Multiple meta-analyses have shown its efficacy in lowering opioid requirements, reducing pain scores, and decreasing hospital length of stay after abdominal operations [18–21]. Similar efficacy has been demonstrated in randomized controlled trials and meta-analyses of utilization in thoracic and breast operations [22–25].

Starting in 2012, our institution implemented an option for physicians to order systemic IVL as a non-opioid adjunct analgesic for patients with rib fractures. We aimed to assess whether this new pain management approach was associated with decreased opioid usage. We hypothesized that patients who received IVL would have lower hospital opioid utilization and similar pain scores as matched patients who did not receive IVL.

## Methods

### Study population

Using our institution’s trauma registry and electronic medical records (EMR) from 2012–2017, we retrospectively evaluated adult patients (age ≥18) admitted to our Level I trauma center with isolated chest wall injuries (Abbreviated Injury Scale, AIS chest ≥3 and all other AIS other body regions <3). Of note, injury severity of trauma patients is quantified using the Injury Severity Score (ISS): ISS = A^2^+B^2^+C^2^, where A, B, and C, are AIS of the three most severely injured body regions. AIS for the six body regions range from 1 (minor) to 6 (unsurvivable). Stanford’s Institutional Review Board approved this study and waived informed consent requirement for this retrospective EMR study.

### Exclusion criteria

Patients were excluded if they had 1) chronic opioid dependence (defined by diagnosis on the patient problem list), 2) rib fractures due to cardiopulmonary resuscitation, 3) presentation to the hospital greater than 24 hours after injury, or 4) discharge from the emergency department without inpatient admission as determined by EMR review.

### Lidocaine protocol

IVL infusion was started at 1mg/kg/hr. Systemic lidocaine levels were measured every 8 hours (therapeutic range 1.5–5 mcg/mL). While bolus dose of 1–1.5 mg/kg is standard practice for perioperative IVL, bolus dose was removed from rib fracture IVL protocol in favor of longer time to therapeutic level (several hours) and mitigating side effects. IVL infusion rate was titrated either by the surgical intensive care unit (SICU) or pain management service (for patients not in the SICU) for adequate analgesia (NRS ≤4) within therapeutic plasma levels. Trained nursing staff and SICU or pain management service monitored potential side effects, which include but are not limited to bradycardia, arrhythmia, numbness, metallic taste, dizziness, or headaches. All patients on IVL had visible bedside sign indicating they were on IVL infusion.

### Measures

#### Exposure variable

IVL infusion (yes/no) was the main explanatory variable. Patients were assigned to the IVL group if they received any lidocaine over the course of their stay.

#### Outcomes variables

The outcomes of interests were: mean daily opioids administered during the hospitalization, total opioids administered in the last 24 hours of hospitalization, and mean 0–10 numeric rating scale (NRS) pain score measured from 24–48 hours after admission. Opioid administration data (via oral, intravenous, patient controlled analgesia (PCA), or patch) was aggregated from EMR and converted to total oral morphine equivalents (OME) using established conversion factors [26]. We excluded palliative morphine infusion for dyspnea and neuraxial opioids (high variability in systemic permeability and OME conversion factors) [27]. Daily opioid administration was calculated as the total OME over the hospitalization period divided by the total number of days hospitalized. Opioids administered in the last 24 hours of hospitalization was the total sum of OMEs in the that time. The mean NRS pain score was calculated as the average pain score of all measurements taken within a range of 24–48 hours from admission.

#### Additional clinical information

Additional information captured from the EMR and trauma registry included patient age, gender, smoking status, ISS, mechanism of injury, number of rib fractures, presence of pulmonary contusion, and presence of scapular, clavicular or sternal fractures. We assessed hospitalization characteristics including hospital length of stay (LOS), SICU admission, incidence of pneumonia, discharge disposition, 30-day readmission rates, and mortality during hospitalization.

### Data analysis

Descriptive statistics were calculated within study groups, including frequencies, proportions, medians and interquartile ranges. We assessed the trend of IVL use within the study population over time in one-year intervals using the Mann-Kendall test (“Kendall” package in R) [28].

### Propensity score models

To account for the potential treatment selection bias in administration of IVL, we performed propensity score (PS) matching between patients who received IVL and those who did not. We applied a 1:1 optimal matching model without replacement, matching for age, number of rib fractures, initial pain score (mean NRS pain score during the first 6 hours of admission), presence of pulmonary contusion, and presence of scapular, clavicular or sternal fractures. Matching was performed using the “Matchit” package in R [29].

### Statistical analysis

Data analysis was conducted using the PS-matched data. Categorical variables were analyzed using chi-square tests (if expected cell sizes ≥ 5) or Fisher’s exact tests (if any expected cell size < 5), and continuous variables were analyzed using Mann-Whitney U tests due to nonparametric distributions of OME measures and pain scores. Simple and multivariable linear regression models were calculated using the “lm()” function from the “stats” R package (three study outcomes: mean daily OME, total last 24 hours OME, and mean NRS) [30]. Primary predictive variables in the models included receipt of IVL, age category (<65 vs. ≥65 years), and the interaction effect of age on receipt of IVL (age category x IVL), while adjusting for ISS and hospital LOS as covariates. As the OME outcome measures demonstrated moderate skewness and kurtosis, we performed sensitivity analyses to recalculate linear regression models after applying square-root transformations of mean daily OME and total OME in the last 24 hours. All analyses were conducted using R [30]. Statistical significance was assessed at the level of alpha = 0.05.

## Results

### Study sample

**Fig 1:**
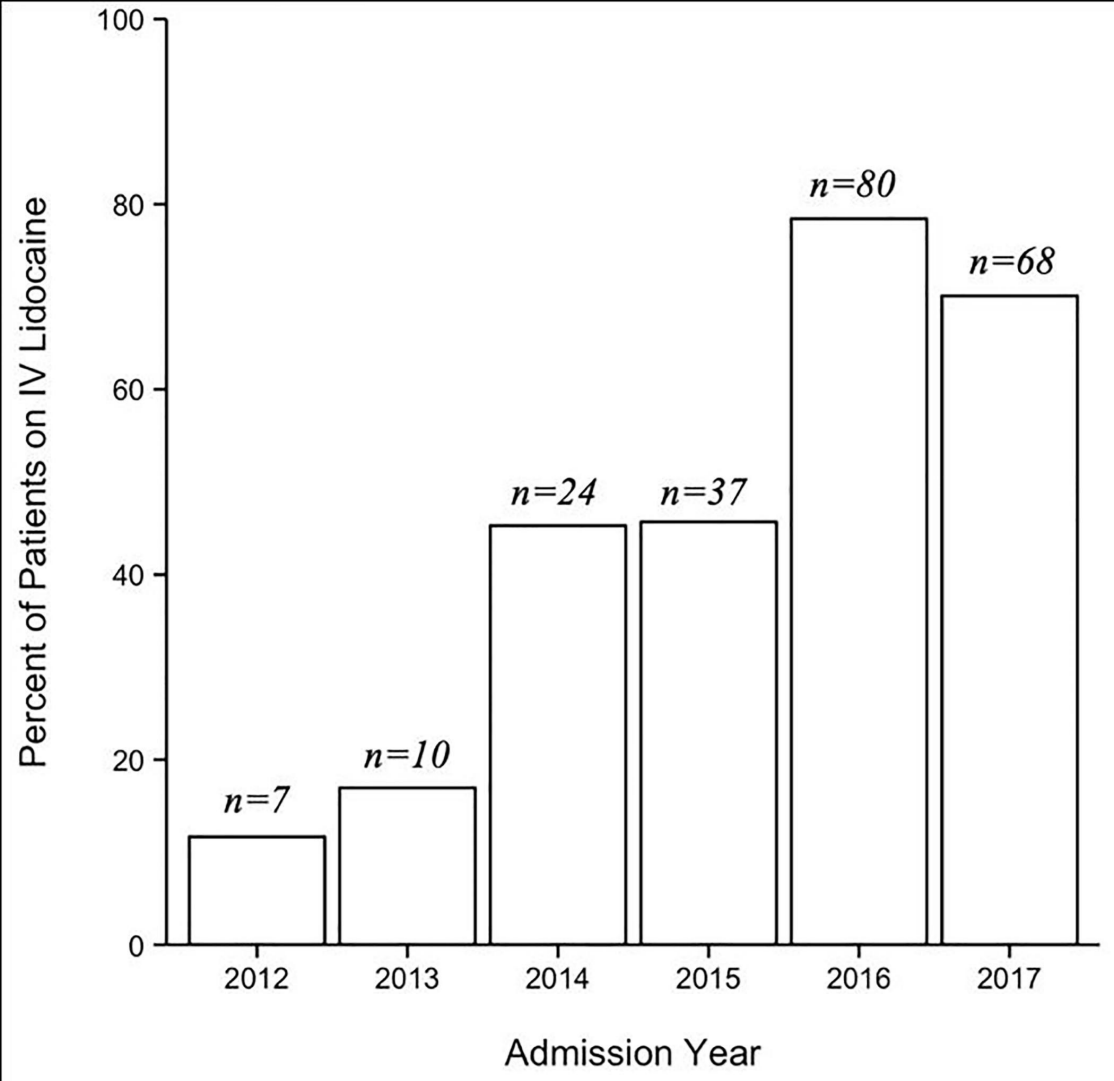
Increase in intravenous lidocaine use over the study period.

During the 6-year study period, we identified 534 adult patients with isolated chest wall injuries in our trauma registry. Among those, 42.3% (n = 226) received IVL. Demographic, injury, and hospitalization characteristics are presented in Table 1. The IVL group was older than the non-IVL group (median age: 67.5 vs 54.5 years, p<0.001). IVL administration was initiated a mean (±SD) of 11.3 (±21.6) hours after admission and infused for a mean (±SD) duration of 55.9 hours (±53.6). The proportion of patients receiving IVL increased throughout our study period from 9.1% in 2012 to 63.6% in 2017 (p = 0.002) (Fig 1). Compared to the non-IVL group, patients who received IVL had more serious injuries (ISS > 15: IVL 32.3% vs non-IVL 20.8%, p = 0.006) and greater number of rib fractures (median: IVL 6 vs non-IVL 5, p<0.001). The rates of pulmonary contusion (36.3% vs 39.3%, p = 0.54) and other fractures (sternal: 8.0% vs 9.4%, p = 0.667; clavicular: 13.7% vs 14.9%, p = 0.79; scapular: 11.1% vs 13.3%, p = 0.52) were not significantly different between IVL and non-IVL groups. The most common mechanisms of injury were ground level fall (25.7%) and motor vehicle collision (24.8%) for the IVL group, and motor vehicle collision (26.6%) and bicycle accident (21.4%) for the non-IVL group. Compared to the non-IVL group, the IVL group had longer hospital LOS (median LOS: 5 vs 3 days, p<0.001), were more likely to be admitted to ICU (76.1% vs 34.7%, p<0.001), and more likely to be discharged to a skilled nursing facility (25.2% vs 9.7%, discharge disposition p<0.001). 30-day readmission rates were similar between the IVL and non-IVL groups (2.2% vs 1.3%, p = 0.50), but the IVL group had higher rates of pneumonia (6.6% vs 0.3%, p<0.001) and mortality (3.1% vs 0.6%, p = 0.04). No patient in the IVL group had supratherapeutic lidocaine plasma levels or hemodynamically significant side effects.

**Table 1:** Demographic, injury, and hospitalization characteristics of adult isolated chest wall injury patients on vs not on IVL infusion.

	IVL	No IVL	
	Median (IQR) or Number (%)	Median (IQR) or Number (%)	p
	n = 226	n = 308	
**Demographic characteristics**			
Age	67.5 (56.3–81.0)	54.5 (41.8–65.0)	<0.001
Gender			0.009
Male	141 (62.4%)	226 (73.4%)	
Female	85 (37.6%)	82 (26.6%)	
Smoking status			<0.001
Active	28 (12.4%)	58 (18.8%)	
Former	47 (20.8%)	42 (13.6%)	
Never	143 (63.3%)	161 (52.3%)	
Unknown	8 (3.5%)	47 (15.3%)	
**Injury characteristics**			
Injury severity score (ISS)			0.006
Moderate (ISS 9–15)	153 (67.7%)	244 (79.2%)	
Major (ISS 16–25)	67 (29.6%)	61 (19.8%)	
Severe (ISS 26–74)	6 (2.7%)	3 (1.0%)	
Unsurvivable (ISS = 75)	0 (0%)	0 (0.0%)	
Mechanism of injury			<0.001
Motor vehicle collision	56 (24.8%)	82 (26.6%)	
Motorcycle collision	27 (11.9%)	50 (16.2%)	
Pedestrian vs auto	7 (3.1%)	11 (3.6%)	
Bicycle accident	25 (11.1%)	66 (21.4%)	
Ground level fall	58 (25.7%)	37 (12.0%)	
Fall from height	43 (19.0%)	42 (13.6%)	
Other	10 (4.4%)	20 (6.5%)	
Injuries			
Number of rib fractures	6 (3–6)	5 (4–7)	<0.001
Sternal fracture	18 (8.0%)	29 (9.4%)	0.67
Clavicular fracture	31 (13.7%)	46 (14.9%)	0.79
Scapular fracture	25 (11.1%)	41 (13.3%)	0.52
Pulmonary contusion	82 (36.3%)	121 (39.3%)	0.54
**Hospitalization characteristics**			
Length of stay	5 (3–8)	3 (2–4)	<0.001
Intensive care unit admission	172 (76.1%)	107 (34.7%)	<0.001
Pneumonia	15 (6.6%)	1 (0.3%)	<0.001
Discharge disposition			<0.001
Home	125 (55.3%)	245 (79.5%)	
Skilled nursing facility	57 (25.2%)	30 (9.7%)	
Rehab	4 (1.8%)	4 (1.3%)	
Acute care hospital	32 (14.2%)	23 (7.5%)	
Other	8 (3.5%)	6 (1.9%)	
30-day readmission	5 (2.2%)	4 (1.3%)	0.50
Mortality	7 (3.1%)	2 (0.6%)	0.04

IVL = intravenous lidocaine, IQR = interquartile range.

### Propensity score matching analysis

After propensity score matching, all 226 patients in the IVL group were matched with 226 of the non-IVL group (73.3% of 304 total non-IVL group). Overall, the matching improved balance between the two groups by 48.1%. However, some imbalance remained with significant differences between IVL and non-IVL groups on certain matching variables (Table 2). Compared to the matched non-IVL group, IVL group patients were older (median age: 67.5 vs 59.5, p<0.001), had higher rates of serious injury (ISS > 15: 32.3% vs 19.5%, p = 0.006) and more rib fractures (median: 6 vs 5, p = 0.006).

**Table 2:** Baseline characteristic comparison of propensity score-matched patients on vs not on IVL infusion.

	IVL	No IVL Median (IQR) or Number (%) n = 226	
	Median (IQR) or Number (%) n = 226		p
Age	67.5 (56.3–81.0)	59.5 (53.0–71.0)	<0.001
Injury severity score (ISS)			0.006
Moderate (ISS 9–15	153 (67.7%)	182 (80.5%)	
Major (ISS 16–25)	67 (29.6%)	42 (18.6%)	
Severe (ISS 26–74)	6 (2.7%)	2 (0.9%)	
Unsurvivable (ISS = 75)	0 (0%)	0 (0%)	
Injuries			
Number of rib fractures	6 (4–7)	5 (4–7)	0.006
Sternal fracture	18 (8.0%)	21 (9.3%)	0.74
Clavicular fracture	31 (13.7%)	33 (14.6%)	0.89
Scapular fracture	25 (11.1%)	32 (14.2%)	0.40
Pulmonary contusion	82 (36.3%)	80 (35.4%)	0.92
NRS pain score, admission hour 0–6	4.5 (2.4–6.0)	4.0 (2.5–6.2)	0.74

IVL = intravenous lidocaine, IQR = interquartile range, NRS = numeric rating scale.

### Main results

Unadjusted comparisons demonstrated no significant differences in daily opioid use between IVL and non-IVL groups; however opioid use in the last 24 hours of admission was 40% lower in the IVL group (p = 0.002, Table 3). There were no observed differences between IVL and non-IVL groups in NRS pain score during admission hours 24–48. When stratified by age category (≥65 vs <65 years), there were no significant differences between IVL and non-IVL groups in any primary outcome (Table 3).

**Table 3:** Primary outcome comparison of patients on vs not on IVL infusion; all propensity score-matched patients and patients stratified by age subgroups.

	All Patients			Age <65 years			Age ≥65 years		
	IVL median (IQR)	No IVL median (IQR)	p	IVL median (IQR)	No IVL median (IQR)	p	IVL median (IQR)	No IVL median (IQR)	p
	n = 226	n = 226		n = 102	n = 148		n = 124	n = 78	
OME per day, mg	65.5 (24.2–120.6)	67.3 (32.6–116.6)	0.40	108.8 (70.6–169.1)	97.6 (57.6–129.0)	0.09	33.2 (13.8–66.9)	29.8 (11.2–54.3)	0.33
OME last 24 hours of admission, mg	45.0 (11.3–105.0)	75.0 (24.0–142.5)	0.002	97.5 (45.0–135.0)	110.5 (52.5–159.0)	0.14	22.3 (3.8–52.6)	24.0 (0.0–60.0)	0.99
NRS pain score, admission hours 24–48	3.3 (2.0–4.9)	3.4 (2.1–4.6)	0.70	4.3 (2.7–5.2)	3.8 (2.8–5.2)	0.69	2.5 (1.4–4.3)	2.2 (1.3–3.5)	0.28

Abbreviations: IVL = intravenous lidocaine, IQR = interquartile range, OME = oral morphine equivalents, NRS = numeric rating scale.

Simple and multivariable linear regression results estimating the effect of IVL on daily opioid use, opioid use in the last 24 hours of admission, and NRS pain score during admission hours 24–48, adjusting for age category (≥65 vs <65 years) as a moderating factor, and controlling for HLOS and ISS are presented in Table 4. In univariate analyses, there was no significant effect of IVL on the outcomes of interest. Similarly, in multivariable regression analyses, there was no main effect of IVL on OME/day (p = 0.47), nor an interaction with age (p = 0.90). Age was significantly associated with OME/day, with elderly patients receiving, on average, 64.2 fewer OME/day (95% CI [-87.53, -40.92], p<0.001). OME in the last 24 hours had similar results, with no significant IVL effect (p = 0.53) or IVL by age interaction (p = 0.38) but a main effect of age, with older participants receiving fewer OMEs in that time period (β = -78.31, 95% CI [-103.98, -52.95], p<0.001). Finally, average NRS pain scores did not significantly differ by IVL nor was the IVL by age interaction effect significant, but we observed a significant main effect of age (β = -1.44, 95% CI [-1.92, -0.95], p<0.001), such that the older patients reported lower pain scores.

**Table 4:** Linear regression results estimating IVL associations with OME and pain measures: Unadjusted estimates (univariate) and multivariable estimates adjusted for injury severity score, hospital length of stay, and age (interaction).

	OME per day, mg		OME last 24 hours of admission, mg		NRS pain score, admission hours 24–48	
	β [95% CI]	p	β [95% CI]	p	β [95% CI]	p
IVL, unadjusted	-0.68 [-17.4, 16.0]	0.94	-12.16 [-30.3, 6.0]	0.19	-0.08 [-0.43, 0.26]	0.63
IVL, adjusted	7.94 [-13.7, 29.6]	0.47	-7.55 [-31.4, 16.3]	0.53	-0.11 [-0.56, 0.35]	0.64

Abbreviations: IVL = intravenous lidocaine, OME = oral morphine equivalents, CI = confidence interval, NRS = numeric rating scale.

### Sensitivity analyses: Transformed OME outcomes

Similar to the primary analyses, we observed significant main effects of age on OME outcomes in sensitivity analyses. There were no significant differences in OME/day by IVL group in univariate or multivariable analyses. Unlike the primary analysis, the transformed OME in the last 24 hrs was significantly lower in IVL patients vs. non-IVL (p = 0.02) in univariate analysis. However, the difference was no longer significant in multivariable analysis (p = 0.08) when adjusted for covariates (age, age x IVL, HLOS and ISS).

## Discussion

In propensity-matched analyses of adult rib fracture patients with isolated chest wall injury, pain management with IVL was associated with a 40% lower opioid utilization in the last 24 hours of hospitalization in unadjusted comparisons. To the best of our knowledge, our study is the first to evaluate an analgesic associated with reduced opioid use for rib fracture patients during the last 24 hours of admission. The crude association between IVL and decreased opioid use in the last 24 hours of admission is particularly interesting given this time period’s association with opioid use at 90 days post-discharge, and possibly, post-discharge opioid prescribing patterns. Clinicians may be more likely to prescribe post-discharge opioids based on requirements during the last days, rather than early days, of admission.

However, the association between IVL and decreased opioid use in the last 24 hours of admission was not seen when stratified by age group (<65 vs ≥65 years). In both adjusted and unadjusted analyses, IVL was not significantly associated with opioid use during hospitalization or pain scores during admission hours 24–48. Our findings show conflicting evidence and only partially corroborate previous studies that showed an association between IVL use and decreased opioid utilization after various operations [18–24]. Notably these studies assessed opioid utilization at different time points after surgery, ranging from 6 hours to up to 72 hours [23, 24].

The retrospective nature of our study has inherent limitations. The decision to prescribe IVL was based on clinician judgement. Although we performed propensity score matching based on clinical factors that may influence whether a patient receives IVL, there remained some differences between matched IVL and non-IVL groups that may be better controlled in a randomized trial. The IVL group patients tended to be older, with more severe injuries, longer hospitalizations, were more likely to be admitted to the SICU, and more likely to be discharged to a skilled nursing facility. This partially reflects our institutional practice, wherein any patient 65 or older with 2 or more rib fractures is initially monitored in the SICU. Additionally, IVL may have been pre-emptively administered for patients expected to have worse pain or higher opioid utilization, leading to a selection of older and sicker patients in the IVL group. We also limited our study cohort to adults with isolated chest wall injuries and did not investigate the impact of IVL for poly-trauma patients with rib fractures. Lastly, our clinical team ensures adequate analgesia among rib fracture patients with deep inspiration and coughing, but we could not delineate NRS pain scores recorded for specific activities. Studying NRS pain scores during standardized activities (i.e. minimum incentive spirometry inspiration volume) may unveil associations between IVL and pain scores among patients with rib fractures.

Future investigations evaluating the efficacy of IVL among patients with rib fractures will need adequate statistical power to detect differences within stratified age groups (<65 and ≥65 years). Having shown opioid-sparing effect in other surgical populations, the efficacy of IVL for other subgroups within the trauma population, such poly-trauma patients with rib fractures, may be considered. Further investigations are also needed to uncover the underlying mechanisms of IVL’s long-lasting analgesic effects.

## Conclusion

Our findings suggest IVL requires further investigation for patients with traumatic rib fractures; IVL was crudely associated decreased opioid requirements in the last 24 hours of admission, the time period associated with opioid use at 90 days post-discharge. However, given the lack of observed IVL effects on opioids when stratified by age group and on multivariable adjusted analyses, we are currently conducting a randomized controlled trial to further evaluate the opioid-sparing effects of IVL. IVL is easy to deliver, easy to titrate, and has relatively few contraindications; its analgesic effect is known to last long-term beyond the duration of infusion. In addition to potentially minimizing opioid utilization during admission through multimodality pain management, we must continue investigating analgesic strategies to decrease opioid utilization after hospitalization.
